# Alzheimer’s disease, dementia, and stem cell therapy

**DOI:** 10.1186/s13287-017-0567-5

**Published:** 2017-05-12

**Authors:** Thomas Duncan, Michael Valenzuela

**Affiliations:** Regenerative Neuroscience Group, Brain and Mind Centre & Sydney Medical School, University of Sydney, Sydney, NSW 2050 Australia

**Keywords:** Alzheimer’s disease, Embryonic stem cells, Induced pluripotent stem cells, Mesenchymal stem cells, Neural stem cells

## Abstract

Alzheimer’s disease (AD) represents arguably the most significant social, economic, and medical crisis of our time. Characterized by progressive neurodegenerative pathology, AD is first and foremost a condition of neuronal and synaptic loss. Repopulation and regeneration of depleted neuronal circuitry by exogenous stem cells is therefore a rational therapeutic strategy. This review will focus on recent advances in stem cell therapies utilizing animal models of AD, as well as detailing the human clinical trials of stem cell therapies for AD that are currently undergoing development.

## Background

Approximately 50 million people live with dementia, with the estimated global cost of care being US$818 billion. As age is the predominant risk factor and national demographics are rapidly ageing, this figure is set to rise to 132 million people by 2050 [1]. Dementia is a fatal clinical disorder characterised by amnesia, progressive cognitive impairment, disorientation, behavioural disturbance, and loss of daily function; Alzheimer’s disease (AD) is the most common associated pathology. It can be argued that dementia is one of the most significant social, economic, and medical challenges of our time.

Less than 5% of AD cases are familial, caused by highly penetrant autosomal mutations of the *PSEN1*, *PSEN2*, and, less frequently, *APP* genes. The majority of AD cases are late onset and sporadic, with established risk factors beyond age including cardiovascular disease, low education, depression, and the *apolipoprotein-E4* (*ApoE4*) gene. Sporadic AD is accordingly of multifactorial origins, driven in part by a complex genetic profile and in part by interacting and intersecting environmental exposures.

It should therefore not be surprising that AD pathology is diverse. Four core features can be discerned. Firstly, tau, an intracellular microtubule-associated protein within neurons important for structural support and axonal transport, becomes hyperphosphorylated, leading to microtubule collapse and aggregation into neurofibrillary tangles. Secondly, sequential cleavage of the APP protein by β- and γ-secretase enzymes leads to extracellular accumulation and aggregation of beta amyloid (Aβ) protein fragments, visible as amyloid plaques in the AD brain. Many pharmacological approaches have attempted to promote amyloid clearance by vaccination [2] and decrease production via secretase inhibition [3]. However, results from human clinical trials indicate that amyloid pathology does not correlate with clinical symptoms and therefore may not be a therapeutically relevant target. The third core feature of AD is the presence of activated microglia, the resident macrophages of the central nervous system (CNS), and found in close association with amyloid plaques. Present from the early stages of the disease, their numbers then decline in the advanced AD brain. Activated microglia produce cytokines, such as tumour necrosis factor (TNF)-α, interleukin (IL)-1β, and nitric oxide (NO), that may exacerbate or attenuate neuroinflammation [4]. Mass neuronal and synaptic loss represents the forth core feature of AD and is the closest correlate of cognitive decline in early AD [5]. AD-related neurodegeneration in the temporal lobe follows a distinct pattern. The entorhinal cortex is first affected, then progressing to the subiculum and CA1 hippocampal subregion and basal forebrain networks. Atrophy of these brain regions and the hippocampus overall co-vary with verbal episodic memory deficits in AD patients [5]. In later stages of the disease neurodegeneration spreads throughout the temporal lobes, eventually affecting most cortical layers. The precise temporal sequencing of this complex admixture of pathologies in human sporadic AD is the subject of intense debate.

Due to the progressive nature of AD, if a stem cell therapy is to be successful it must target a well-defined clinical subset of patients. Given the involvement of hippocampal circuitry in the early phases of the disease, we suggest this region as a potential therapeutic target. There is now an enormous global demand for new effective therapies that not only halt progression but also reverse symptoms. In this review, we argue that a potentially effective strategy is to target the biological feature most closely tied to symptoms, namely neurosynaptic loss. Specifically, we focus on recent advances in cell-based therapies that aim at repopulation or regeneration of degenerating neuronal networks in AD.

### Stem cell classes

An important step in developing any stem cell therapy is to choose the appropriate cell source. The most commonly utilized cells in recent AD studies are embryonic stem cells (ESCs), mesenchymal stem cells (MSCs), brain-derived neural stem cells (NSCs), and induced pluripotent stem cells (iPSCs). ESCs are derived from the inner cell mass of the developing blastocyst (at embryonic day 5 to 6) and are classified as pluripotent because they possess the ability to generate cell types from the ectodermal, mesodermal, and endodermal germ layers. MSCs are involved in the development of mesenchymal tissue types and can be harvested from umbilical cord blood (UCB-MSCs) or Wharton’s jelly, and also remain present in several adult stem cell niches including bone marrow and adipose tissue. Classified as multipotent, MSCs are able to generate multiple cell types that share a common embryonic origin, namely the mesodermal germ layer. Despite this, phenotypic expression and the differentiation potential of MSCs can vary according to the tissue of origin [6]. Similarly multipotent, NSCs are responsible for the generation of all neural cell types during development. While also present in the adult brain, they are restricted to the discrete neurogenic niches of the subventricular zone and the granular layer of the dentate gyrus in the hippocampus. Finally, iPSCs are derived from mature somatic cells in vitro, commonly adult dermal fibroblasts, and are genetically modified by small molecule treatment or viral vector-delivered transcription factor upregulation to become pluripotent and ESC-like in phenotype and differentiation capacity [7].

### Endogenous repair

There are several theoretical approaches to the design of a stem cell therapeutic strategy for early AD. One is to target upregulation of resident NSC niches within the adult brain, in effect stimulating adult hippocampal neurogenesis to compensate for neurodegeneration. Adult hippocampal neurogenesis may have a key role in learning and memory, and so promoting this process may help counter the amnestic symptoms of early AD. One option has been to upregulate (pharmacologically or with gene therapy) those growth factors known to positively regulate neurogenesis, including brain-derived neurotrophic factor (BDNF), insulin growth factor-1 (IGF-1), nerve growth factor (NGF), and vascular endothelial growth factor (VEGF) [8].

This approach is, however, complicated by several quantitative challenges. Firstly, the rate of hippocampal neurogenesis decreases with age in humans, with an estimated 800 new neurons produced daily in adulthood declining to ~100 in late life under disease-free conditions. Since the best estimates suggest neuronal number is stable in normal ageing, this is therefore the minimum required to achieve neuronal equilibrium because of rapid neuronal turnover. Secondly, in AD there is mass loss of hippocampal neurons. In the dentate gyrus the loss is estimated at ~1 M, and in CA1 the loss is estimated at ~5 million. Hence, to compensate for AD there would need to be an order-fold increase in hippocampal neurogenesis to normalise dentate gyrus numbers. Furthermore, adult hippocampal neurogenesis has no effect whatsoever on CA1 neurons and so the main neuronal deficit in early AD is unaddressed. Third, this approach must account for the effect of AD pathology on neurogenesis, for which there is conflicting evidence from animal studies [9, 10]. Overall, endogenous strategies for neuronal repair in early AD lack potency and miss one of the main neuronal targets.

### Exogenous cell therapy

Exogenous cell therapies aim to restore degenerate neuronal networks, and consequently cognitive function, through the introduction of stem cells. These stem cells may be used as a cellular delivery system, utilizing a paracrine “bystander” mechanism through either native or induced production of neuroprotective growth factors. Alternatively, therapeutic restoration may occur through differentiation and participation of the stem cells in repopulating degenerate neuronal circuits. This is a finely balanced, complex, and multistep process. Each class of stem cells has different propensities to achieve these approaches, as briefly reviewed here. Details of recent AD model stem cell transplantation studies featured in this review are summarized in Table 1.

**Table 1:** AD rodent model stem cell transplantation studies in the last 5 years

Study	[23]	[24]	[26]	[27]	[34]	[35]	[36]	[37]	[38]	[39]	[45]
Cell type	Murine embryonic NSCs	Human fetal NSCs	Human fetal NSCs	Human fetal NSCs	Human UCB-MSCs	Human PD-MSCs	Human U-MSCs Human U-MSC-NCs	Human A-MSCs	Murine BM-MSCs	Human BM-MSCs	Human iPSC-derived neuronal precursors
Model	B6C3-Tg (APPswe/PSEN1dE9) transgenic mice	NSE-APPswe transgenic mice	Tg2576 (APPswe) transgenic mice	3×Tg-AD transgenic mice CaM/Tet-DT_A_ mice	APP/PS1 transgenic mice	Aβ_1–42_ cerebrally infused mice	B6C3-Tg (APPswe/ PSEN1dE9) transgenic mice	Tg2576 (APPswe) transgenic mice 3xTg-AD transgenic mice	APP/PS1 transgenic mice	Aβ_1–42_ cerebro-ventricular infused mice	PDAPP transgenic mice
Delivery route	Bilateral intra-hippocampal stereotactic injection 5 × 10^5^ to 1 × 10^6^ cells *Sham*: PBS vehicle	Bilateral intra-ventricular stereotactic injection 5 × 10^5^ cells *Sham*: H-H buffer vehicle	Bilateral intra-hippocampal stereotactic injection 2.5 × 10^5^ cells *Sham*: culture media vehicle	Bilateral intra-hippocampal stereotactic injection 1 × 10^5^ cells *Sham*: vehicle	Three bilateral intra-hippocampal injections at 2 week intervals 1 × 10^5^ cells per injection *Sham*: PBS vehicle	Intravenous injection 1 × 10^5^, 5 × 10^5^, or 1 × 10^6^ cells *Sham*: Saline vehicle	Bilateral intra-hippocampal stereotactic injection 5 × 10^4^ cells *Sham*: PBS vehicle	Intravenous injection 2 × 10^6^ cells *Sham*: PBS vehicle	Intravenous injection 1 × 10^6^ cells *Sham*: NaCl solution vehicle	Intravenous injection 1 × 10^6^ cells *Sham*: PBS vehicle	Bilateral intra-hippocampal stereotactic injection 2 × 10^5^ cells *Sham*: PBS vehicle
Findings	*10 weeks post-operation* Extensive donor cell migration 14.6% neuron, 36.2% astrocyte, and 28.5% oligodendroctye phenotypic differentiation Improved spatial memory (Morris water maze) Decreased expression of pro-inflammatory cytokines IL-1β, IL-6, TNF-α and PGE2 Aβ levels unchanged	*7 weeks post- operation* Extensive donor cellular migration NSC phenotype remained in >80% of cells Improved spatial memory (Morris water maze) Decreased levels of phosphorylated tau, Aβ plaques, astrogliosis, microgliosis and apoptosis Decreased expression of pro-inflammatory cytokines IL-1β, IL-6, TNF- α and iNOS Increased cerebral neurotrophin levels and increased hippocampal synaptic density	*5 weeks post- operation* Donor cells in the dentate gyrus polymorphic layer 70% neuron, 20% astrocyte phenotypic differentiation Improved spatial memory (Morris water maze) Increased endogenous neurogenesis in the dentate gyrus Reduced cerebral Aβ levels	*6 weeks post-operation* Donor cells in the CA1 hippocampal subregion 36.6% and 41.1% cell survival in 3 × Tg-AD and CaM/Tet-DT_A_, respectively Improved spatial memory (Morris water maze, context- and place-dependent NOR task) Majority of donor cells expressed NSC phenotype Increased levels of synaptic proteins in the hippocampus Soluble, insoluble and hyperphosphorylated tau, Aβ_40_, and Aβ_42_ levels unchanged	*41 days post-operation (first injection)* Improved spatial memory (Morris water maze) Reduced phosphorylated tau, Aβ plaques, vascular Aβ_40_, and BACE-1 expression in the cortex and hippocampus Increased levels of activated microglia in the cortex and hippocampus Reduced levels of pro-inflammatory cytokines IL-1β and TNF-α, and increased anti-inflammatory cytokine IL-4	*2 weeks post-operation* Limited donor cells in the hippocampus, and no neural differentiation Improved spatial memory (Morris water maze) Reduced levels of cerebral APP and BACE1, and reduced β- and γ-secretase activity Reduced levels of activated astrocytes and microglia Attenuation Aβ_1–42_ induced hippocampal apoptosis, and impaired endogenous neuronal differentiation Reduced expression of inflammatory proteins iNOS and COX-2, and an array of pro-inflammatory cytokines	*4 weeks post-operation* No donor cells present at 4 weeks post-surgery Improved spatial memory (Morris water maze) in the U-MSC-NC group Increased hippocampal levels of synapsin I in the U-MSC-NC group Decreased hippocampal Aβ deposition, decreased soluble Aβ_40_ and Aβ_42_ levels, and increased Aβ-degrading enzymes in the U-MSC-NC group Increased number of M2 activated microglia in the U-MSC-NC group Reduced pro-inflammatory cytokines (IL-1β and TNF-α), and increased anti-inflammatory cytokine IL-4 in the U-MSC-NC group	*6 weeks post-operation (Tg2576 mice)* Improved spatial memory (Morris water maze) *1 and 12 weeks post-operation (3 × Tg-AD mice)* Donor cells in the spleen, lung, liver, but not brain Reduced number and size of Aβ plaques Increased density of activated microglia in the hippocampus by week 1, lower density than in sham animals by week 12 Increased phagocytotic microglia Reduced proinflammatory cytokines IL-1 and TNF-α at week 1 Increased anti-inflammatory cytokines IL-10 and TNF-β at week 12 Increased levels of Aβ-degrading enzymes	*1 and 4 -weeks post-operation* Donor cells in the cerebral cortex and hippocampus, bone marrow, lung, and liver No reduction in total Aβ levels Reduced total levels and vascular deposition of pE3-Ab protein at 4 weeks Increased number of <50 μm Aβ plaques, and reduced number of 50–100 μm Aβ plaques Reduced levels of activated astrocytes and ramified microglia Reduced levels of cortical and hippocampal microglia Reduced levels of hippocampal TNF-α, IL-6, and elevated levels of hippocampal PTGER2	*1, 2, and 4 weeks post-operation* Donor cell neuronal differentiation in the entorhinal cortex and hippocampus Improved working memory performance (Radial Arm Maze) Attenuation of impaired neurogenesis and neuronal differentiation in the hippocampus at 2- and 4-week time points Increased hippocampal expression of neural specification proteins β-catenin and Ngn1	*2 weeks post-operation* Improved spatial memory (Morris water maze) *45 days post-operation* Improved spatial memory (Morris water maze) Donor cell survival and neuronal differentiation in the hippocampus Donor cells expression of cholinergic and GABAergic neuronal markers
Therapeutic mechanism	Modulation of inflammation	Modulation of inflammation and microglia immune response, and protection from Aβ neurotoxicity	Neurotrophic support of endogenous neurogenesis and synaptic connectivity	Neurotrophic support of endogenous neurogenesis and synaptic connectivity	Modulation of inflammation and microglia, and anti-amyloidogenic	Neurotrophic support of endogenous neurogenesis, modulation of inflammation and microglia immune response, and anti-amyloidogenic	Modulation of inflammation and microglia immune response	Modulation of inflammation and microglia immune response	Modulation of microglia immune response	Neurotrophic support of endogenous neurogenesis and protection from Aβ neurotoxicity	Regeneration of depleted neural networks

*Aβ* amyloid beta, *AD* Alzheimer’s disease, *A-MSC* adipose-derived mesenchymal stem cell, *BM-MSC* bone marrow-derived mesenchymal stem cell, *COX* cyclooxygenase, *GABA* gamma-aminobutyric acid, *H-H* Henderson-Hasselbalch, *IL* interleukin, *iNOS* inducible nitric oxide synthase, *iPSC* induced pluripotent stem cell, *Ngn* neurogenin, *NOR* novel object recognition, *NSC* neural stem cell, *PBS* phosphate-buffered saline, *PD-MSC* placenta-derived mesenchymal stem cell, *PGE* prostaglandin, *PTGER* prostaglandin E receptor, *TNF* tumour necrosis factor, *U-MSC* umbilical cord Warton’s jelly-derived mesenchymal stem cell, *U-MSC-NC* neuron-like cell differentiated from umbilical cord Warton’s jelly-derived mesenchymal stem cell, *UCB-MSC* umbilical cord blood-derived mesenchymal stem cell

#### ESCs

While some ESC transplantation studies have shown a capacity to restore cognitive function in rodent models of brain injury [11], their clinical translation has been limited. This is in part due to their pluripotent nature, as transplantation of undifferentiated ESCs presents an inherent risk of uncontrolled cell growth and tumour formation [12]. In vitro pre-differentiation of ESCs into NSCs circumvents some of this risk, generating predominantly cholinergic neurons and inducing improvements in spatial memory performance after transplantation into an AD rodent model [13]. More recently, one study reported the stable generation of cholinergic neuronal populations from human ESCs which, following transplantation, were able to functionally integrate into hippocampal neuronal circuitry [14]. In 2013, another study reported the conversion of ESCs into medial ganglionic eminence-like progenitor cells—a transient stem cell type present in the developing brain. Following transplantation into a murine brain injury model, these cells were capable of maturing into both GABAergic and cholinergic neuronal subtypes and synaptically integrating with host neuronal circuits, leading to improvements in impaired spatial memory and learning [15]. Despite ongoing preclinical studies, there are inherent ethical and immunogenic limitations to using allogeneic donor cells that significantly hamper the clinical translation of ESC-based therapies.

#### NSCs

The paracrine effect of NSCs has been shown to have significant therapeutic potential. Transplanting growth factor-secreting NSCs increased neurogenesis and cognitive function in a rodent AD model [16] and aged primate brain [17], while transplantation of choline acetyltransferase-overexpressing human NSCs into a cholinergic neurotoxic rodent model resulted in a reversal of spatial memory and learning deficits [18]. Other recent AD rodent model studies have reported that NSC transplantation decreased neuroinflammation [19], attenuation of tau and Aβ AD neuropathology [20], promotion of neurogenesis and synaptogenesis [21, 22], and reversal of cognitive deficits [19, 21, 22]. While the therapeutic mechanisms behind these changes are not yet fully understood, they are likely mediated by both the paracrine release of neuroprotective or immune modulatory factors [16] and by direct neuronal differentiation [13, 23], although the widespread generation of non-neuronal glial cell types from transplanted NSCs remains a major limiting factor for neuroreplacement strategies [23].

#### MSCs

Due to their accessibility, relative ease of handling, and the broad range of cell types that they are able to generate, MSCs are now among the most frequently studied stem cell type. In aged rodent models, transplanted MSCs were shown to undergo differentiation into neural cell types, increasing local concentrations of acetylcholine neurotransmitter, BDNF, and NGF, and improving locomotor and cognitive function [24]. However, to date there has been little evidence for the functional or synaptic maturation of MSC-derived neurons in vivo. Moreover, genuine neuroreplacement by MSCs remains limited by low rates of neuronal differentiation and a propensity for glial cell formation in vivo [25]. Potentially of greater therapeutic significance are the reported neuroprotective paracrine effects of MSCs, with the introduction of MSC-secreted factors able to stimulate proliferation, neuronal differentiation, and survival in endogenous neurogenic niches [26, 27] and in cellular models of AD [28]. Similarly, in rodent AD models, MSC transplantation has been reported to inhibit Aβ- and tau-related cell death [28, 29], reduce Aβ deposits and plaque formation [30–33], stimulate neurogenesis, synaptogenesis, and neuronal differentiation [28, 31, 34], and rescue spatial learning and memory deficits [29–32]. Some studies suggest a further anti-inflammatory and immune modulatory paracrine effect for transplanted MSCs, including upregulated neuroprotective cytokines such as IL-10, and reduced levels of pro-inflammatory cytokines TNF-α and IL-1β [29–32]. Intravenously administered MSCs are also capable of crossing the blood-brain barrier and effectively migrating to regions of neural injury, without inducing a tumourigenic or immune response [35]. This minimally invasive approach has significant advantages over traditional intracranial injection when considering human clinical translation, although reports of MSCs infiltrating into multiple organs remains a concern for this delivery system [34, 35].

#### iPSCs

iPSC-derived neurons are structurally and functionally mature, and capable of forming electrophysiologically active synaptic networks [36]. Using additional transcription factors during the induction process, it has also been possible to direct differentiation into specific neuronal subtypes, such as dopaminergic neurons [37]. As iPSCs are a relatively new technology, preclinical animal model transplantation studies are few. One study in an ischaemic stroke rodent model demonstrated that human iPSC-derived NSCs were able to improve neurological function and reduce pro-inflammatory factors through a neurotrophin-associated bystander effect [38]. In another recent study, following intra-hippocampal transplantation into a transgenic AD mouse model, human iPSC-derived cholinergic neuronal precursors survived, differentiated into phenotypically mature cholinergic neurons, and reversed spatial memory impairment [39].

iPSC technology allows for the production of autologous pluripotent stem cells, thereby avoiding both the ethical limitations and immune rejection issues of non-patient-specific sources. Long-term survival and efficacy of autologous iPSC-derived dopaminergic neuronal transplantation has been demonstrated in a simian Parkinson’s disease model, with improved motor activity and function, and extensive cell survival and engraftment at 2-years post-operation [40]. However, autologous iPSCs may be of limited use for neuroreplacement as neurons generated from AD patients display phenotypic neuropathology, including abnormal Aβ levels, elevated tau phosphorylation, reduced neurite length, and altered electrocompetency [41–43]. Alternatively, using iPSC-derived neurons to recapitulate AD pathology in vitro has significant applications in the study of pathogenesis and screening for potential therapeutic drugs. As such, they are now the subject of extensive study in vitro, as reviewed elsewhere [44].

### Stem cell trials in humans

Inconsistencies in preclinical studies have prevented several potential stem cell therapies from transitioning to human clinical trials. By contrast, evidence for the safety and efficacy of MSC-based therapies in animal models, combined with ease of handling and isolation, has supported the approval of several human clinical trials.

A recently completed open-label phase I clinical trial evaluated the safety and the tolerability of intracranially injected allogeneic human umbilical cord blood-derived MSCs (Trial identifier: NCT01297218, NCT01696591) [45]. Nine patients, defined by the National Institute of Neurological and Communicative Disorders and Stroke-Alzheimer’s Disease and Related Disorders Association criteria as having probable AD, were enrolled in the trial. Mini-Mental State Examination scoring between 10 and 24 (mild-moderate AD dementia), and Pittsburgh compound B positron emission tomography confirmation of Aβ pathology were used as inclusion criteria. Trial participants were then divided into low-dose (3 × 10^6^ cells; *n* = 3) and high-dose (6 × 10^6^ cells; *n* = 6) groups, and received bilateral stereotactic injection of human umbilical cord blood-derived MSCs into the hippocampus and precuneus. At 3 months and 24 months post-treatment time points, no patient showed any serious adverse event resulting from either the surgical procedure or transplantation of MSCs. However, MSC transplantation did not slow cognitive decline over the 24 months of follow-up, as measured by the Alzheimer’s Disease Assessment Scale-cognitive subscale. Furthermore, no changes to AD pathology were observed. The neuroprotective effect of MSCs, frequently reported in AD animal models [30–32], was therefore not evident. The authors suggest this may be due in part to a reliance on neuroimaging rather than more sensitive post-mortem biochemical analyses used in animal studies.

Details of ongoing trials are summarised in Table 2. While many of these employ an intravenous infusion administration route, one trial (Trial identifier: NCT02054208) will assess the safety and efficacy of intraventricular MSC injection via an Ommaya reservoir system. Umbilical cord blood-derived MSCs remain a common cell choice, although key differences exist with regards to cell number, dose number, and dose schedule. Two separate trials, both currently undergoing recruitment, will utilise alternative MSC sources. One trial (Trial identifier: NCT02912169) will assess the safety and efficacy of autologous adipose-derived stromal vascular fraction cells acquired from patient liposuction. Another study (Trial identifier: NCT02833792) will utilise ischaemia-tolerant allogeneic human bone marrow-derived MSCs. Grown under hypoxic conditions to more closely resemble the physiological environment of the CNS, these MSCs express higher levels of angiogenic growth factors, including VEGF and angiopoietin, and show enhanced migratory activity [46].

**Table 2:** Ongoing stem cell trials in humans with Alzheimer’s disease

Trial ID	NCT01547689	NCT02054208	NCT02600130	NCT02912169	NCT02833792	NCT02672306	NCT02899091
Date	03/2012 to 12/2016	02/2014 to 02/2018	11/2015 to 10/2019	11/2015 to 12/2017	06/2016 to 06/2018	05/2016 to 10/2019	09/2016 to 06/2018
Study design	Phase I/II Safety and efficacy Intervention Single group open-label	Phase I/II Safety and efficacy Intervention Randomized Double-blind Placebo-controlled	Phase I Safety and efficacy Intervention Randomized Double-blind Placebo-controlled	Phase I/II Safety and efficacy Intervention Non-randomized Single group Open-label Multi-centre	Phase II Safety and efficacy Intervention Randomized Single-blind Placebo-controlled Multi-centre	Phase I/II Safety and efficacy Intervention Randomized Double-blind Placebo-controlled	Phase I/II Safety and efficacy Intervention Randomized Double-blind Placebo-controlled
Stage	Active	Recruiting	Recruiting	Recruiting	Recruiting	Not yet recruiting	Not yet recruiting
Cell type	hUCB-MSCs	hUCB-MSCs	hBM-MSCs	hAD-SVF	hBM-MSCs	hUCB-MSCs	hPD-MSCs
Inclusion criteria	Age 50–85 Probable AD K-MMSE 3–20	Age 50–85 Probable AD K-MMSE 18–26 Amyloid^+^ PIB/florbetaben-PET	Age 50–80 Diagnosed AD K-MMSE 18–24 Amyloid^+^ PET	Age ≥55 Probable AD (NINCDS-ADRDA and DSM IV)	Age 55–80 Mild-moderate AD K-MMSE 12–24 Amyloid^+^ florbetapir-PET	Age 50–85 Probable AD K-MMSE 3–20	Age ≥50 Probable AD K-MMSE 10–26 Amyloid^+^ PET
Delivery route	Intravenous infusion	Ommaya Reservoir intraventricular injection	Intravenous infusion	Intravenous and intranasal infusion	Intravenous infusion	Intravenous infusion	Intravenous infusion
Arms	*n* = 30 Eight infusions once every 2 weeks in the first month of each quarter 2 × 10^7^ cells per infusion	*n* = 42 Three injections at 4-week intervals *Low-dose group:* 1 × 10^7^ cells per injection *High-dose group:* 3 × 10^7^ cells per injection *Placebo group:* saline	*n* = 30 Single infusion *Low-dose group:* 2 × 10^7^ cells *High-dose group:* 1 × 10^8^ cells *Placebo group:* Plasmalyte A and 1% human serum albumin	*n* = 100 Single intravenous infusion or intravenous and intranasal infusion	*n* = 40 Single infusion Crossover at 6 months post-infusion *Group 1:* 1.5 × 10^6^ cells/kg bodyweight *Group 2:* lactated Ringer’s Solution	*n* = 40 Eight infusions at 2-week intervals *Treatment group:* 2 × 10^7^ cells per infusion *Placebo group:* saline	*n* = 24 Single or repeat (day 0 and week 4) infusions *Arm 1:* K-MMSE 20–26 *Arm 2:* K-MMSE 10–19 *Group1:* 2 × 10^8^ cells *Group 2:* two infusions of 2 × 10^8^ cells *Placebo group:* placebo infusion
Outcome measures	*10 weeks FU* No. of adverse events Change from baseline: ADAS-cog, MMSE, CIBIC, ADCS-ADL and CGA-NPI CSF transthyretin, Aβ and tau Blood Thl/Th2 cytokines	*24 weeks FU* No. of adverse events Change from baseline: ADAS-cog, S-IADL, K-MMSE, CIBIC, CGA-NPI and CDR CSF biomarkers MRI DTI mapping, PIB-PET and FDG-PET	*30 days FU* No. of adverse events *2, 4, 13, 39, and 52 weeks FU* Change from baseline: ADAS-cog, MMSE, CGA-NPI and GDS CSF inflammatory markers, Aβ and tau Blood inflammatory and AD biomarkers MRI brain volumetry	*12 months FU* No. of adverse events *3 and 6 months FU* Change from baseline: FAQ, GDS, MMSE and ADCS-ADL	*18 months FU* No. of adverse events Change from baseline: Neurological examinations	*10 weeks FU* No. of adverse events Change from baseline: ADAS-cog, MMSE, ADCS-CCGIC, ADCS-ADL and CGA-NPI CSF Aβ and tau Blood Aβ	*48 weeks FU* No. of adverse events Change from baseline: ADAS-cog, K-MMSE, GDS, CDR, K-IADL, CGA-NPI, CIBIC and SF-36 CSF Aβ and tau Brain MRI, amyloid-PET, FDG-PET, CMRglc Quantitative ECG

*Aβ* amyloid beta, *AD* Alzheimer’s disease, *ADAS-cog* Alzheimer’s Disease Assessment Scale-Cognitive Subscale, *ADCS-ADL* Alzheimer’s Disease Cooperative Study Activities of Daily Living, *ADCS-CCGIC* Alzheimer’s Disease Cooperative Study Clinician’s Global Impression of Change, *CDR* Clinical Dementia Rating, *CGA-NPI* Caregiver-administered Neuropsychiatric Inventory, *CIBIC* Clinician’s Interview-Based Impression of Change, *CMRglc* cerebral metabolic rate for glucose, *CSF* cerebrospinal fluid, *DSM* Diagnostic and Statistical Manual of Mental Disorders, *DTI* diffusion tensor imaging, *ECG* electrocardiogram, *FAQ* Functional Activities Questionnaire, *FDG* fluorodeoxyglucose, *FU* follow-up, *GDS* Geriatric Depression Scale, *hAD-SVF* human adipose-derived stromal vascular fraction, *hBM-MSC* human bone marrow-derived mesenchymal stem cell, *hPD-MSC* human placenta-derived mesenchymal stem cell, *hUCB-MSC* human umbilical cord blood-derived mesenchymal stem cell, *K-IADL* Korean Instrumental Activities of Daily Living, *K-MMSE* Korean version of Mini-Mental State Evaluation, *MMSE* Mini-Mental State Evaluation, *MRI* magnetic resonance imaging, *NINCDS-ADRDA* National Institute of Neurological and Communicative Disorders and Stroke and the Alzheimer's Disease and Related Disorders Association, *PET* positron emission tomography, *PIB* Pittsburgh compound B, *SF-36* 36-item Short Form Health Survey, *S-IADL* Seoul-Instrumental Activities of Daily Living, *Th* T helper

### Future directions

Preclinical studies suggest that stem cells have potential for the treatment of AD; however, this area is notable for poor translation between animal studies and human trials. Indeed, researchers have effectively treated AD in transgenic mouse models in more than 50 different ways [47]. Transgenic models demonstrate little, if any, predictive utility. Their outcomes are frequently model-dependent and, disappointingly, each approach has failed in human clinical trials. Transgenic models are largely based on familial AD-related hypotheses in a genetically homogeneous population, while the vast majority of human AD occurs sporadically amongst a distinctly heterogeneous population. Moreover, they do not recapitulate the extensive neuronal and synaptic loss that is central to AD. Clearly, rodent models and their aetiological hypotheses are inadequate for predicting human clinical outcomes. AD cell therapies will therefore need to demonstrate success in higher-order animals that more faithfully mimic the clinical and neurodegenerative features of the human condition.

Several key questions also need to be addressed, including long-term safety, optimum cell source and the delivery system, understanding donor cell response to the pathogenic AD environment, and clarifying the mechanisms of action. Many of the studies discussed here utilised inherently heterotopic stem cells. While this is a clinically relevant strategy due to the inaccessible nature of the adult NSC niche, this too requires careful consideration. Human and rodent studies have reported tumour formation resulting from autologous haematopoietic stem cell [48], allogeneic fetal NSC [49], and genetically engineered MSC [50] transplantation. While neuroreplacement therapies may not be able to fully compensate for widespread and progressive neuronal loss, they may serve to temporarily enhance existing depleted circuits, which is sufficient to improve cognition function, restore daily function, and improve quality of life. Upon diagnosis, lifespan for individuals with AD dementia is 4–5 years, and so if a neuroreplacement therapy could rescue and protect brain function for that timespan it is commensurate to a functional cure. Alternatively, due to the complex nature of AD pathophysiology, a multimodal approach may be required, incorporating pharmacological targeting of pathology, stimulation of endogenous neurogenesis and synaptogenesis, as well as exogenous neuroreplacement.

## Conclusion

Stem cell therapy for AD carries enormous promise but remains under development. There is now substantive preclinical literature that demonstrates proof-of-concept, with new studies continuing to reveal potential therapeutic mechanisms. MSC-based therapeutics have been the most consistent and have reached human clinical trials. To date, one such trial was negative but there are many others underway. Researchers must, however, be aware of the perilous gulf that lies between rodents and humans. Not only do we need to better understand the cells and the brains they intend to repair, but also employ translational models that begin to bridge this gap.

## Abbreviations

Aβ: Amyloid beta
AD: Alzheimer’s disease
ApoE4: Apolipoprotein-E4
BDNF: Brain-derived neurotrophic factor
CA: Cornu Ammonis
CNS: Central nervous system
ESC: Embryonic stem cell
GABA: Gamma-aminobutyric acid
IGF-1: Insulin growth factor-1
IL: Interleukin
iPSC: Induced pluripotent stem cell
MSC: Mesenchymal stem cell
NGF: Nerve growth factor
NO: Nitric oxide
NSC: Neural stem cell
TNF: Tumour necrosis factor
UCB-MSC: Umbilical cord blood-derived mesenchymal stem cell
VEGF: Vascular endothelial growth factor
